# A GhBGH2‐GhGLK1 Regulatory Module Mediates Salt Tolerance in Cotton

**DOI:** 10.1111/pbi.70300

**Published:** 2025-08-06

**Authors:** Peilin Wang, Jiamin Wang, Huan Si, Chenhui Li, Lili Zhou, Xinyue Xu, Weilong Li, Xiaofeng Su, Wenfang Guo, Xifeng Chen, Bojun Ma, Huiming Guo, Hongmei Cheng

**Affiliations:** ^1^ Academician Workstation National Nanfan Research Institute, Chinese Academy of Agricultural Sciences Sanya China; ^2^ National Key Laboratory of Agricultural Microbiology Biotechnology Research Institute, Chinese Academy of Agricultural Sciences Beijing China; ^3^ College of Life Science Zhejiang Normal University Jinhua China; ^4^ Tobacco Research Institute Chinese Academy of Agricultural Sciences Qingdao China; ^5^ Yazhouwan National Laboratory Sanya China

**Keywords:** cotton, *GhBGH2*, GhGLK1 transcription factor, salt tolerance, transcriptional activity

## Abstract

Soil salinisation, exacerbated by climate change and human activities such as irrigation mismanagement, improper land use and excessive fertilisation, has become a major constraint on global crop production by disrupting fundamental metabolic processes like seed germination and photosynthesis. In our previous work, transcriptome sequencing of salt‐tolerant and salt‐sensitive cotton germplasms identified *Gh_D04G136300*, a negative regulatory gene downregulated in salt‐tolerant and salt‐sensitive materials. Phylogenetic analysis revealed its closest homologue to be *AtBGH2*, leading to its designation as *GhBGH2*. Virus‐induced gene silencing (VIGS) demonstrated that *GhBGH2* silencing enhanced salt tolerance. To further validate its function, we generated *bgh2* knockout mutants via CRISPR/Cas9, which exhibited increased salt tolerance compared to controls. Transcriptome sequencing and yeast two‐hybrid screening identified GhGLK1 as an interacting protein. Both GhBGH2 and GhGLK have nuclear localisation. Functional characterisation through VIGS revealed that GhGLK1 positively regulates salt tolerance in cotton. Yeast one‐hybrid (Y1H), dual‐luciferase (LUC) and electrophoretic mobility shift assays (EMSA) confirmed that GhGLK1 binds to G‐box elements in the promoters of downstream salt‐tolerance genes, activating their transcription. Structural analysis of GhGLK1 revealed a transcriptional activation domain at its C‐terminus, and yeast heterologous expression along with co‐immunoprecipitation (Co‐IP) assays demonstrated that GhBGH2 interacts with this domain. Haplotype analysis of GhGLK1 identified a distinct *Hap‐1* variant enriched in China's northwestern saline‐alkali regions. This variant exhibited elevated GhGLK1 expression and conferred enhanced salt tolerance. Collectively, our findings indicate that GhBGH2 negatively regulates salt tolerance in cotton by interacting with the GhGLK1 activation domain, suppressing its transcriptional regulation of salt‐tolerance genes.

## 1 Introduction

With the intensification of climate change, crops must adapt to increasingly diverse and challenging environmental conditions that hinder their growth and development (Malhi et al. 2020). These adverse conditions encompass biotic and abiotic stresses, with drought, salinity and extreme temperatures being the primary environmental factors restricting plant distribution, limiting crop yields and threatening global food security (Zhu 2016). Saline‐alkali soils, characterised by excessive soluble salts, are formed through complex interactions involving climate, geography, hydrology and anthropogenic activities such as unsustainable farming practices (Liang et al. 2024). Soil salinisation is a major abiotic stressor severely restricting agricultural productivity, particularly in irrigated regions. Over 45 million hectares of irrigated land are affected by salinity, significantly reducing crop yields and jeopardising sustainable agricultural production amid rising food demand (Quesada et al. 2000; Shabala and Cuin 2008).

Cotton ( *Gossypium hirsutum* L.) is a globally significant cash, fibre and oil crop. Although relatively salt‐tolerant, high salinity impairs seed germination and overall plant development (Leidi and Saiz 1997; Saghir et al. 2002). As arable land diminishes due to environmental changes, cotton cultivation increasingly shifts to saline and arid regions. Salt tolerance varies across different developmental stages, with germination being particularly sensitive—high salinity inhibits germination, reducing seedling establishment rates (Higbie et al. 2010). Although cotton exhibits greater salt tolerance during vegetative growth, excessive salinity suppresses transpiration, impairs photosynthesis and water uptake efficiency and elevates respiration rates (Sharif et al. 2019). Phenotypically, salinity stress reduces plant height, arrests leaf expansion and compromises stem, shoot and root growth. Prolonged exposure further stunts fruit development and degrades fibre quality (Gupta and Huang 2014; Chaudhary et al. 2024).

The *Brz‐insensitive‐pale green* (*bpg*) gene family regulates chloroplast function downstream of brassinosteroid (BR) signalling and comprises *BPG1*, *BPG2* and *BPG3* (Tachibana et al. 2022; Komatsu et al. 2010; Yoshizawa et al. 2014). *BPG1* encodes the 3,8‐divinyl protochlorophyllide, an 8‐vinyl reductase (*DVR*) enzyme, which is essential for chlorophyll biosynthesis (Nagata et al. 2005). *BPG2* encodes a highly conserved chloroplast GTPase critical for rRNA maturation (Tachibana et al. 2022). *BPG4 Homologous Gene 2 (BGH2)* is homologous to *BPG4* and is implicated in chloroplast development (Tachibana et al. 2024).

Golden2‐like (GLK) transcription factors belong to the GARP (Golden2, ARR‐B, Psr1) superfamily, which consists of three subfamilies. Most *GLK* genes contain two conserved domains: a Myb‐DNA binding domain (which includes a helix–loop–helix motif) and a C‐terminal domain (with a conserved GCT‐box) (Kreps et al. 2002; Tamai et al. 2002). GLK transcription factors were initially identified as key regulators of chloroplast development, and they are now known to upregulate genes associated with the photosynthetic light system, thereby promoting chloroplast function (Nakamura et al. 2009). In *Arabidopsis*, the *GLK* promoter contains elements related to plant hormones, photosynthesis, and stress responses. *AtGLK* gene members are involved in metal ion transport and contribute to drought, cold, and osmotic stress tolerance (Alam et al. 2022). Overexpression of peanut *GLK* in *Arabidopsis glk1*/*glk2* double mutants restores drought resistance to wild‐type levels (Liu et al. 2020). In cotton, *GLK1* silencing results in more severe leaf wilting under cold and drought stress, whereas overexpression of *GLK1* in *Arabidopsis* enhances tolerance to these stressors (Liu et al. 2021).

In our previous work, we conducted transcriptome sequencing on salt‐tolerant and salt‐sensitive cotton germplasms subjected to graded salt stress (Wang, Nie, et al. 2023; Wang, Zhang, et al. 2023). Through screening for negative regulators of salt tolerance, we identified *GhBGH2*, which was downregulated in salt‐tolerant lines and salt‐sensitive lines. Silencing *GhBGH2* enhanced salt tolerance. Using CRISPR, we generated *bgh2* mutant lines that exhibited greater salt tolerance and more developed root systems under salt stress compared to controls. Yeast two‐hybrid screening identified *GhGLK1* as an interacting partner of *GhBGH2*. *GhGLK1* was strongly induced by salt stress and highly expressed under stress conditions, whereas its silencing reduced salt tolerance. Interaction assays demonstrated that GhBGH2 binds to the transcriptional activation domain of *GhGLK1*, repressing downstream salt tolerance genes and rendering plants more sensitive to salt stress. Haplotype analysis revealed a significant mutation in *GhGLK1* among cotton populations in China's saline‐alkali inland regions. Functional studies confirmed that this mutation enhanced *GhGLK1* expression, conferring improved salt tolerance. Overall, our findings elucidate the molecular mechanism by which GhBGH2 negatively regulates salt tolerance through its interaction with GhGLK1, providing a foundation for developing salt‐tolerant cotton germplasms.

## 2 Materials and Methods

### 2.1 Plant Materials Growth Conditions and Salt Stress Treatment

Cotton recipient Z49 (also named CRI49), along with the CRISPR lines *bgh2‐1* and *bgh2‐2*, were cultivated in nutrient‐rich soil in pots under greenhouse conditions with a photoperiod of 16 h light and 8 h darkness, maintaining a temperature of 28°C ± 2°C and a relative humidity of 70%. Plants grown for 3 weeks were used in the experiments. For salt treatment, seeds were treated with water (control) and 200 mM NaCl, and each treatment consisted of 30 seeds with three replications. Seedlings were watered with 400 mM NaCl for 1 week, after which any evidence of wilting was recorded. Leaves were then excised, immediately frozen with liquid nitrogen and stored at −80°C until analysis. In the field, cotton material was planted in the experimental field of the Biotechnology Research Institute, Chinese Academy of Agricultural Sciences (Langfang, Hebei Province and Sanya, Hainan Province).

### 2.2 Determination of Physiological Indicators

Physiological and biochemical indexes are an important aspect of detecting plant resistance to abiotic stress. In this experiment, the contents of superoxide dismutase (SOD), catalase (CAT), hydrogen peroxide (H_2_O_2_) and malondialdehyde (MDA) were determined in leaves of cotton with similar size in the same part after salt stress. Determination of SOD by Solarbio Micro Superoxide dismutase (SOD) Assay Kit: bc0715; CAT by catalase (CAT) Assay Kit: bc0200; H_2_O_2_ Content Assay Kit: bc3595 and MDA Content Assay Kit: bc0025. Each assay was performed in triplicate for each line, and the results were statistically analysed using SPSS software and plotted by Origin software.

### 2.3 RNA Sequencing and Data Analysis

Library construction and sequencing were performed in accordance with the standard experimental procedures provided by the Illumina 2000 system. The differentially expressed genes (DEGs) were identified using DESeq2 (Love et al. 2014) by a |Fold Change| ≥ 1. The resulting *p*‐value was adjusted using the Benjamini and Hochberg's approach. Genes with an adjusted *p*‐value < 0.05 as determined by DESeq2 were assigned as differentially expressed. EggNOG‐mapper v2 (Huerta‐Cepas et al. 2019) was used for gene annotation. Gene Ontology (GO) and Kyoto Encyclopedia of Genes and Genomes (KEGG) pathway enrichment analysis of the DEGs was performed using the cluster profiler R package (Yu et al. 2012).

### 2.4 RNA Extraction, cDNA Preparation and RT‐qPCR Analyses

Total RNA was extracted using the FastPure Plant Total RNA Isolation Kit (Polysaccharides and Polyphenolics‐rich, RC401‐01, Vazyme Biotech, Nanjing, China) and digested with DNase I to eliminate DNA. The HiScript III All‐in‐one RT SuperMix Perfect Kit for qPCR, R333‐01 (Vazyme). Quantitative real‐time PCR was performed on three biological replicates with the ABI 7500 Real‐Time PCR system using the ChamQ Universal SYBR qPCR Master Mix (Q711‐02, Vazyme). Cotton histone h3, which exhibits a stable expression in the different tissues, developmental stages and environmental conditions (Zhu 2016), was used as an internal control. Expression was measured using the 2^−ΔΔCT^ method (Livak and Schmittgen 2001).

### 2.5 Virus‐Induced Gene Silencing Assays

To further study whether the knockdown of GhBGH2 and GhGLK1 affected the salt stress tolerance, we conducted VIGS experiments. Briefly, 400–500 bp fragments of each of the two genes were amplified from cDNA. These recombinant plasmids were transformed into *Agrobacterium tumefaciens* strain LBA4404. The transformed Agrobacterium cells were grown overnight at 28°C. The Agrobacterium cultures containing pTRV1 and pTRV2 or its derivative vectors were mixed at a ratio of 1:1 with helper expression plasmid 192, and plants with two fully expanded cotyledons (but without a true leaf) were infiltrated with the Agrobacterium‐mixed suspension using a needle‐less syringe. The plants were left at room temperature under dim light overnight and subsequently grown at 23°C with a 16 h:8 h, light: dark cycle.

### 2.6 Yeast Two‐Hybrid Assays

GhBGH2 was amplified and cloned into the prey vector pGADT7, and GhGLK1 was amplified and cloned into the bait vector pGBKT7 (to delineate the specific interaction region, GhGLK1 was divided into four fragments: 1–114 aa, 115–230 aa, 231–329 aa and 330–423 aa). A pGBKT7/bait plasmid and a pGADT7/prey plasmid were co‐transfected into the yeast strain Y_2_HGold and verified on selective media DDO (SD/Trp‐Leu) and DDO (SD/Trp‐Leu‐His‐Ade) following the instructions provided for the Matchmaker Gold Yeast Two‐Hybrid System (Beijing Zoman Biotechnology company).

### 2.7 Subcellular Localisation of GhBGH2 and GhGLK1 Proteins

The open reading frames of GhBGH2 and GhGLK1 were inserted into the pCAMBIA1302 vector. This construct was introduced into *Agrobacterium tumefaciens* EHA105 (*pSoup*), which was subsequently transformed to cotton protoplast (Wang et al. 2022). The protoplast was analysed by confocal microscopy (Laser confocal super‐resolution microscope LSM980) with bright field and fluorescence imaging.

### 2.8 Bimolecular Fluorescence Complementation

GhBGH2 was amplified and cloned into pSPYCE, and GhGLK1 was amplified and cloned into the bait vector pSPYNE. This construct was introduced into *Agrobacterium tumefaciens* EHA105 (*pSoup*) and then transformed to cotton protoplast (Wang et al. 2022). The abaxial epidermis of transgenic tobacco was analysed by confocal microscopy (Laser confocal super‐resolution microscope LSM980) with bright field and fluorescence imaging.

### 2.9 Co‐Inmunoprecipitation Assays

For the Co‐ip assays, GhBGH2 was fused with the GFP tag in the pCXDG vector, and GhGLK1 was fused with the HA tag in the pCXSN‐HA vector. Agrobacterium strain GV3101 cells containing individual constructs were co‐infiltrated into *N. benthamiana* leaves. Total proteins were extracted from the samples as described previously. Protein A agarose gel (10279981, GEHealthcare, Sweden) was used for preclearing. Following immunoprecipitation with 3 μg GFP‐specific antibody (ab290, Abcam, China), the beads were washed six to eight times with lysis buffer and collected for immunoblot detection. The fusion protein was detected by immunoblotting using monoclonal anti‐Flag antibody (F1804, Sigma, China) and monoclonal anti‐HA antibody (H9658, Sigma, China).

### 2.10 Electrophoretic Mobility Shift Assay

The coding region of the GhGLK1 was fused in‐frame with GST in the pGEX‐6p‐2 vector and expressed in *Escherichia coli Rosetta* (DE3) cells. The recombinant GhGLK1‐GST protein used for EMSA was purified using GsT‐Sep Glutathione Agarose Resin 4FF (20508ES10, YEASEN, China). The probes were labelled with biotin using an EMSA Probe Biotin Labeling Kit (GS008, Beyotime, China). Recombinant protein was incubated with labelled probes at room temperature for 20 min, and unlabelled probes were used as a competitor to examine the specificity of binding. Protein/promoter fragment complexes were separated by electrophoresis on a native 4% acrylamide gel. The DNA was electroblotted onto nitrocellulose membranes and detected using a Chemiluminescent EMSA Kit (GS009, Beyotime, China). EMSA images were photographed using a Fusion SL4spectral imaging system (Vilber, Beijing, China).

### 2.11 Dual Luciferase Assay

The GhGLK1 full‐length encoding sequence was cloned and inserted into the PGreenII‐62‐SK vector. A G‐box binding element was identified at specific distances from the ATG of all the candidate salt‐related genes. Effector plasmids, reporter plasmids and empty plasmids were introduced into Agrobacterium EHA105 (pSoup). Subsequently, suspensions containing the effector plasmid and reporter plasmid were co mbined in a 1:1 ratio and infiltrated into tobacco leaves, where they were cultured at 25°C for 48–72 h. For assessment, D‐Luciferin potassium salt (10 μM) was sprayed onto the injected areas of the tobacco leaves. Imaging was then conducted using the LB985 Night SHADE fluorescence imaging system (Berthold Technologies, Bad Wildbad, Germany). The transcriptional activity of firefly luciferase and Renilla luciferase (REN) was quantified using a GloMax 20/20 luminometer (Promega, Madison, WI, United States) and the Dual‐Glo Luciferase Assay System according to the manufacturer's instructions. Each sample was measured with six biological replicates.

### 2.12 Statistical Analysis

Statistical analyses were conducted utilising Origin9 software for some analyses and SPSS 22.0 software for others. The data are expressed as mean values ± standard deviation (SD). For comparisons among multiple groups, one‐way analysis of variance (ANOVA) followed by Dunnett's post hoc test were employed. In cases involving only two groups, Student's *t*‐test was utilised for data analysis.

### 2.13 Haplotype Analysis

The 4180 cotton accessions in the CottonMD database were classified into eight genetic groups (G0–G7) based on their genetic background. G0 consisted of wild *G. hirsutum* accessions from the Americas, while G1 included *G. hirsutum* landraces from Mesoamerica. G2 primarily comprised landraces from southern China. G3 predominantly included accessions from Northwest China (NWC) and North China (NC). G4 comprised accessions from three historical cotton‐planting areas in China. G5 contained accessions from the Yangtze River region (YZR). G6 included accessions from the Yangzi River region (YZR) in China and the United States.

## 3 Results

### 3.1 *GhBGH2* Gene Identification, Evolution and Functional Analysis

**FIGURE 1:**
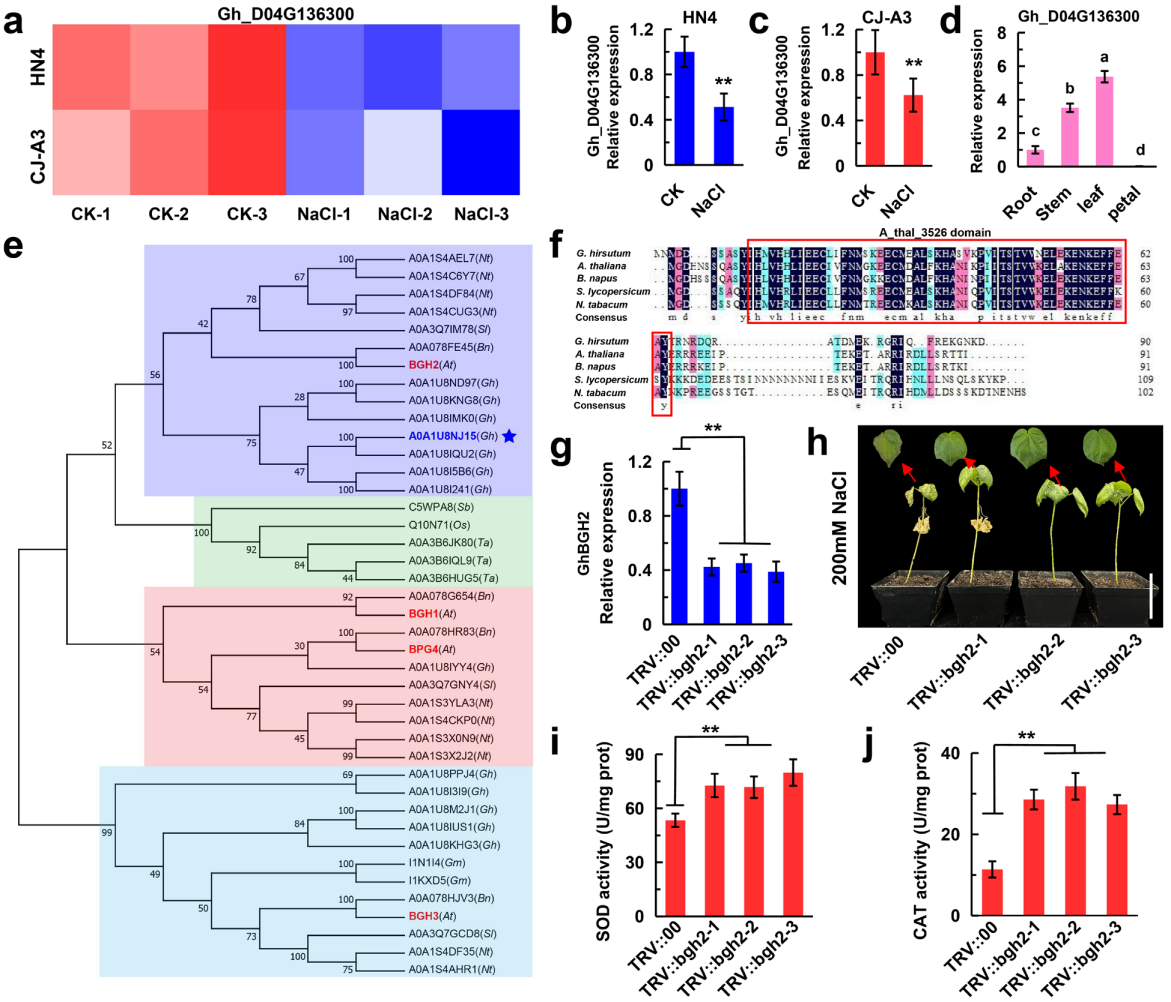
Identification, evolution and functional analysis of *GhBGH2*. (a) Heatmap of *Gh_D04G136300* expression in HN4 and CJ‐A3 under control (CK) and 200 mM NaCl treatments. (b, c) RT‐qPCR analysis of *Gh_D04G136300* expression in HN4 and CJ‐A3 under CK and 200 mM NaCl conditions. Data are presented as mean ± SD (*n* = 3 replicates). **p < 0.01. Student's *t*‐test. (d) RT‐qPCR analysis of Gh_D04G136300 expression across different tissues (root, stem, leaf, petal) in Z49. Data represent mean ± SD (*n* = 3 replicates). *p* < 0.05. ANOVA. (e) Phylogenetic analysis of *Arabidopsis BGH* genes (*At3g55240*, *At3g28990*, *At5g02580*, *At1g10657*) and their homologues in *Gossypium hirsutum* (Gh), *Arabidopsis thaliana* (At), *Brassica napus* (Bn), *Solanum lycopersicum* (Sl) and *Nicotiana tabacum* (Nt). (f) Sequence alignment of *BGH2* homologues, with the *A_thal_3526* domain highlighted in orange. Accession numbers: A0A1U8NJ15 (Gh), Q84TG0 (At), A0A078FE45 (Bn), A0A3Q7IM78 (Sl), A0A1S4CUG3 (Nt). (g) RT‐qPCR analysis of *GhBGH2* transcript levels in TRV::00 and TRV::GhBGH2 cotton. Data are presented as mean ± SD (*n* = 3 replicates). **p < 0.01. Student's *t*‐test. (h) Phenotypic comparison of TRV::00 and TRV::GhBGH2 cotton plants following 200 mM NaCl treatment. Bar = 6 cm. (i, j) SOD and CAT activities in VIGS‐treated plants under 200 mM NaCl stress. Data represent mean ± SD (*n* = 3 replicates). **p < 0.01. Student's *t*‐test.

In previous studies, we selected two upland cotton cultivars, HN4 (salt‐tolerant) and CJ‐A3 (salt‐intolerant), and subjected them to varying NaCl concentrations (100, 150, 200 and 250 mM) at the seedling stage. RNA‐Seq analysis was then performed to identify DEGs associated with salt tolerance (Wang et al. 2024). Through KEGG and GO pathway analyses, we identified several salt‐tolerance–related genes. Among them, we screened for negatively regulated genes and identified *Gh_D04G136300*, downregulated in HN4 and CJ‐A3 under salt stress (Figure 1a). RT‐qPCR validation confirmed that its expression pattern was consistent with transcriptome data (Figure 1b,c). Tissue‐specific expression analysis revealed that *Gh_D04G136300* exhibited the highest expression in leaves (Figure 1d).

As *Gh_D04G136300* was unannotated, we conducted homology alignment with orthologous sequences from cotton, *Arabidopsis thaliana* , rapeseed, tomato and tobacco. Phylogenetic analysis demonstrated that *Gh_D04G136300* clustered most closely with *A. thaliana AtBGH2*, leading us to designate it as *GhBGH2* (Figure 1e). Further sequence analysis revealed that *GhBGH2* harbours a highly conserved A_thal_3526 domain, suggesting functional conservation across species (Figure 1f).

To investigate the functional role of *GhBGH2*, we employed virus‐induced gene silencing (VIGS). RT‐qPCR confirmed effective gene silencing in TRV::*GhBGH2* plants (Figure 1g). Following treatment with 200 mM NaCl, TRV::*GhBGH2* plants exhibited significantly reduced wilting compared to controls, indicating enhanced salt tolerance (Figure 1h). Furthermore, key antioxidant enzymes, superoxide dismutase (SOD) and catalase (CAT), exhibited higher activity in TRV::*GhBGH2* plants under salt stress, suggesting improved oxidative stress resistance (Figure 1i,j).

### 3.2 Knockout of *GhBGH2* Enhances Salt Tolerance in Cotton

**FIGURE 2:**
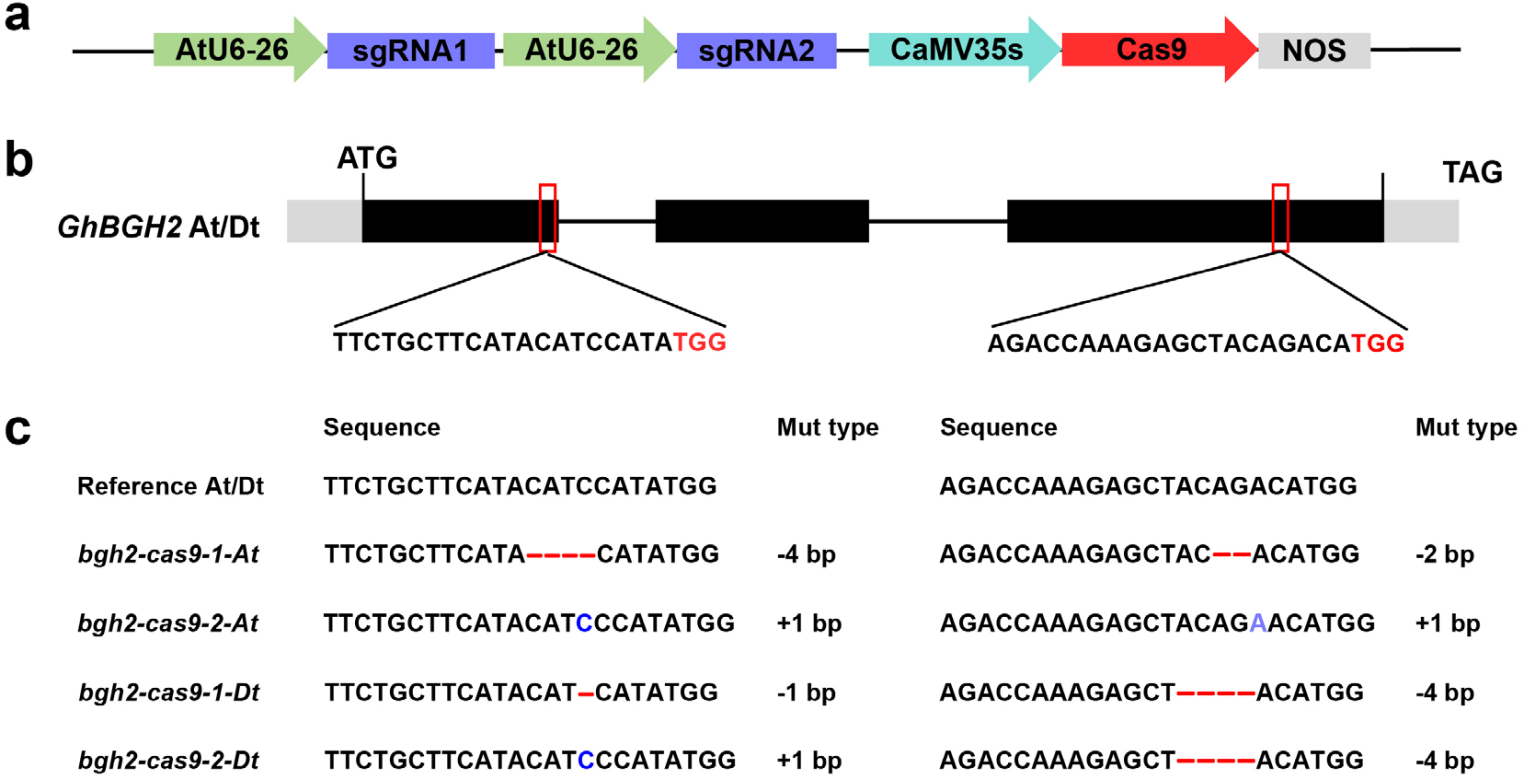
Generation and validation of *bgh2* knockout mutants. (a) Schematic representation of the pCBSG015 vector and sgRNA insertion site. (b) Target site localisation within the *GhBGH2* CDS, showing sequence details. (c) Hi‐TOM sequencing results for *bgh2‐cas9‐1* and *bgh2‐cas9‐2*. Red lines and ‘–’ denote deletions, while blue letters and ‘+’ indicate insertions. At and Dt represent the A‐ and D‐genome, respectively.

To generate *GhBGH2* knockout lines, we designed two sgRNAs targeting exon 1 and exon 3, which were cloned into a Cas9‐sgRNA cassette (Figure 2a,b). Two independent T0 transgenic lines were obtained, and mutation analysis via the Hi‐TOM platform confirmed successful editing in both subgenomes. In *bgh2‐cas9‐1*, the A subgenome exhibited a 4‐bp deletion at sgRNA1 and a 2‐bp deletion at sgRNA2, while the D subgenome had a 1‐bp deletion at sgRNA1 and a 4‐bp deletion at sgRNA2. *Bgh2‐cas9‐2* displayed a 1‐bp insertion at both sgRNA1 and sgRNA2 in the A subgenome, along with a 1‐bp insertion and a 4‐bp deletion at sgRNA2 in the D subgenome (Figure 2c).

**FIGURE 3:**
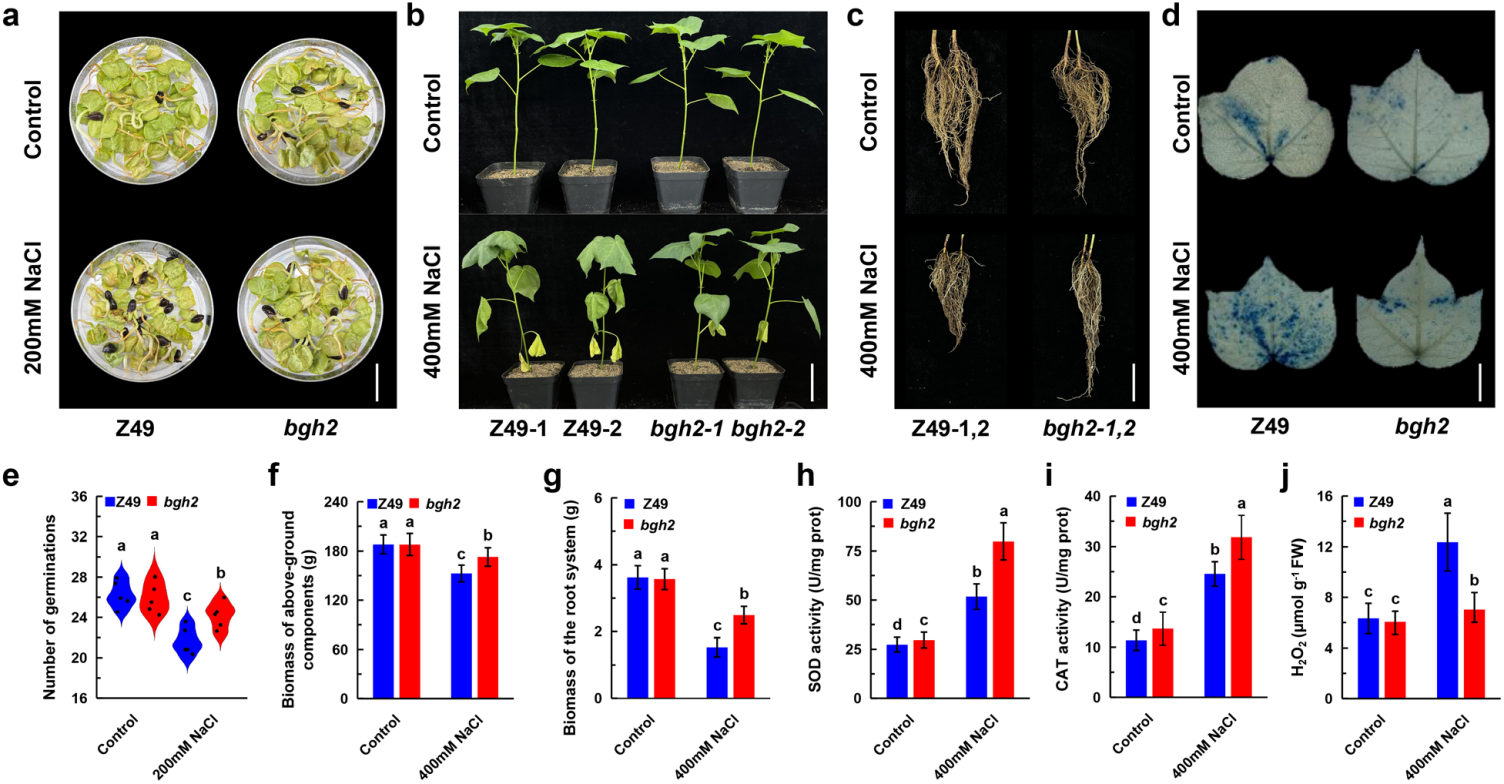
Knockout of *GhBGH2* enhances salt tolerance in cotton. (a) Germination of Z49 and *bgh2* seeds under CK and 200 mM NaCl. Bar = 3 cm. (b) Phenotypic comparison of Z49 and *bgh2* plants under CK and 400 mM NaCl. Bar = 6 cm. (c) Root morphology of Z49 and *bgh2* plants under CK and 400 mM NaCl. Bar = 3 cm. (d) Trypan blue staining of Z49 and *bgh2* leaves under CK and 400 mM NaCl. Bar = 3 cm. (e) Seed germination rate of Z49 and *bgh2* (30 seeds per replicate) under CK and 200 mM NaCl. Data represent mean ± SD (*n* = 3 replicates). *p* < 0.05. ANOVA. Above‐ground biomass (f) and root biomass (g) of Z49 and *bgh2* under CK and 200 mM NaCl. (h–j) SOD, CAT, and H_2_O_2_ levels in Z49 and *bgh2* under CK and 200 mM NaCl. Data are presented as mean ± SD (*n* = 3 replicates). *p* < 0.05. ANOVA.

Given that *GhBGH2* silencing conferred salt tolerance, we further assessed salt tolerance in *bgh2* mutants under 200 mM NaCl. Germination rates were comparable to wild‐type (WT) under normal conditions, but under salt stress, *bgh2* seeds exhibited significantly higher germination rates than the WT control (Figure 3a). Additionally, when 4–6 true‐leaf‐stage seedlings were treated with 400 mM NaCl for 15 days, WT plants showed severe wilting, whereas *bgh2* mutants displayed only mild symptoms (Figure 3b). Root system analysis revealed that *bgh2* mutants had significantly longer roots, more lateral roots and thicker taproots compared to WT under salt stress (Figure 3c). Biomass measurements indicated that both shoot and root biomass were significantly higher in *bgh2* mutants under salt stress (Figure 3d,e). Furthermore, SOD and CAT activities were elevated in *bgh2* mutants, reinforcing their improved salt tolerance (Figure 3f). Based on the salt tolerance identification and analysis of China Cotton Research Institute, the field survival rates of the two lines of *bgh2* under salt tolerance were 0.5799 and 0.7427 (Figure S1), which is a good salt‐resistant material.

### 3.3 Transcriptome Enrichment Analysis Reveals GhBGH2‐Mediated Salt Tolerance

To elucidate the molecular mechanisms underlying GhBGH2‐mediated salt tolerance, we performed transcriptome sequencing on leaves from Z49 (WT), *bgh2‐1* and *bgh2‐2* mutants. A total of 53 905 DEGs were identified in Z49 versus *bgh2‐1*, and 54 112 DEGs in Z49 versus *bgh2‐2*. Filtering for DEGs with |Log_2_FoldChange| ≥ 1 and *p* ≤ 0.05, followed by Venn analysis, revealed 4800 shared DEGs (Figure S2a).

GO enrichment analysis highlighted significant upregulation of pathways associated with plant stress responses, including defence response, oxidative stress response, cellular stress response and oxidoreductase activity. Additionally, pathways related to signal transduction (intracellular signalling, intracellular transport, active transmembrane transport and transmembrane receptor activity) and photosynthesis (Photosystem II, photosynthetic membrane) were enriched (Figure S2b).

KEGG pathway analysis further identified the upregulation of multiple biosynthetic pathways contributing to salt tolerance, such as flavonoid biosynthesis, brassinosteroid biosynthesis, monoterpenoid biosynthesis and diterpenoid biosynthesis. Similar to GO enrichment, pathways involved in signal transduction (MAPK signalling, ABC transporters and the phosphatidylinositol signalling system) and photosynthesis (circadian rhythm regulation, TCA cycle, porphyrin and chlorophyll metabolism) were significantly enriched (Figure S2c).

### 3.4 GhBGH2 Interacts With the Transcriptional Activation Domain of GhGLK1

**FIGURE 4:**
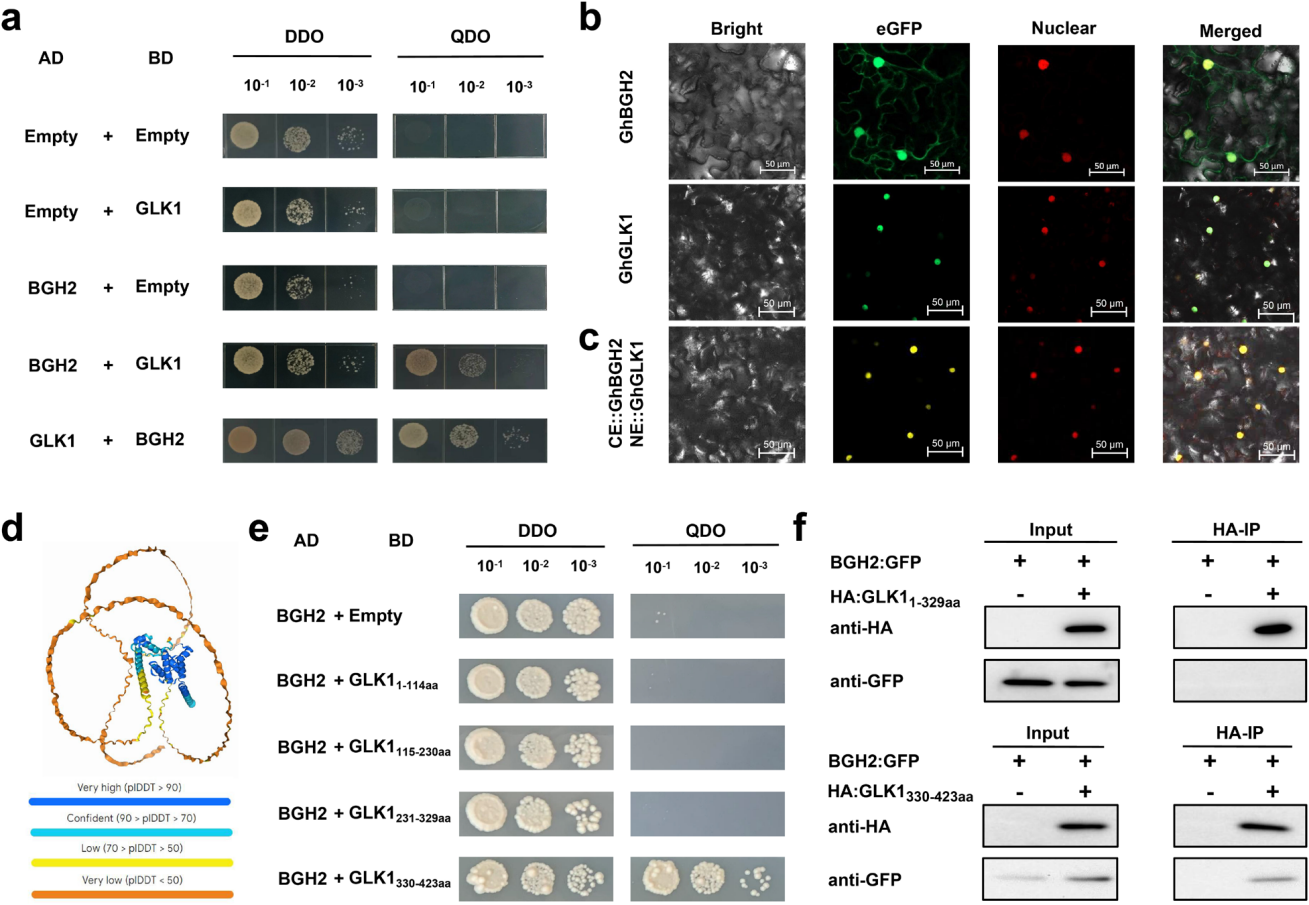
GhBGH2 interacts with the transcriptional activation domain of *GhGLK1*. (a) Y2H assay showing the interaction between GhBGH2 and GhGLK1. Serial dilutions (1×, 10×, 100×) were spotted on DDO and QDO plates. ‘Empty’ denotes an empty AD/BD vector. (b) Subcellular localisation of pCaMV35S::GhBGH2/GhGLK1‐eGFP, with nuclear marker in red. Scale bar: 50 μm. (c) BiFC assay confirming the interaction between CE::GhBGH2 and NE::GhGLK1. Scale bar: 50 μm. (d) AlphaFold3‐predicted GhGLK1 structure, highlighting the interaction region in blue. Local distance difference test (pLDDT) indicates per‐residue confidence. (e) Y2H assay mapping the GhBGH2‐interacting regions within GhGLK1 (divided into four segments: 1–114 aa, 115–230 aa, 231–329 aa, 330–423 aa). Serial dilutions (1×, 10×, 100×) were spotted on DDO and QDO plates. ‘Empty’ denotes an empty BD vector. (f) Co‐IP assay confirming in vivo interaction between GFP‐GhBGH2 and HA‐GhGLK1 (329 aa and 330–423 aa) in *N. benthamiana*. Protein extracts were immunoprecipitated with anti‐GFP and analysed via anti‐GFP and anti‐HA immunoblots.

A yeast two‐hybrid screen, combined with transcriptome sequencing and GO/KEGG pathway analysis, identified the transcription factor GhGLK1 as a potential interactor of GhBGH2. Yeast two‐hybrid assays confirmed that GhBGH2 physically interacts with GhGLK1 (Figure 4a). Subcellular localisation analysis revealed that GhBGH2 is localised to both the nucleus and cell membrane, whereas GhGLK1 is exclusively nuclear (Figure 4b). Further validation using BiFC assays in tobacco confirmed the nuclear interaction of GhBGH2 and GhGLK1 (Figure 4c).

GhGLK1 belongs to the GARP transcription factor superfamily and comprises four distinct regions: an N‐terminal acidic domain, a DNA‐binding domain, a proline‐rich region and a C‐terminal domain containing a conserved GCT‐box, which functions as a transcriptional activation domain (Figure S3a). Sequence alignment of GLK1/2 homologues from *Arabidopsis*, maize, rice and tomato revealed strong conservation in the DNA‐binding and C‐terminal domains, with moderate conservation in the proline‐rich region (Figure S3b). Phylogenetic analysis indicated that GhGLK1/2 is evolutionarily distinct from homologues in other species (Figure S3c).

AlphaFold3‐based structural prediction (alphafoldserver.com) (Abramson et al. 2024) localised the GhBGH2‐GhGLK1 interaction site to the blue structural domain, corresponding to the C‐terminal region of GhGLK1 (Figure 4d and Figure S3d). To delineate the specific interaction region, GhGLK1 was divided into four fragments: 1–114 aa, 115–230 aa, 231–329 aa and 330–423 aa. Yeast two‐hybrid assays demonstrated that GhBGH2 interacts with the GCT‐box in the C‐terminal region (Figure 4e). This was further corroborated by co‐immunoprecipitation (Co‐IP), where GhBGH2 associated with GhGLK1_330‐423aa_ but not GhGLK1_1‐329aa_, confirming the interaction with the GCT‐box domain (Figure 4f).

### 3.5 GhGLK1 Enhances Salt Tolerance

**FIGURE 5:**
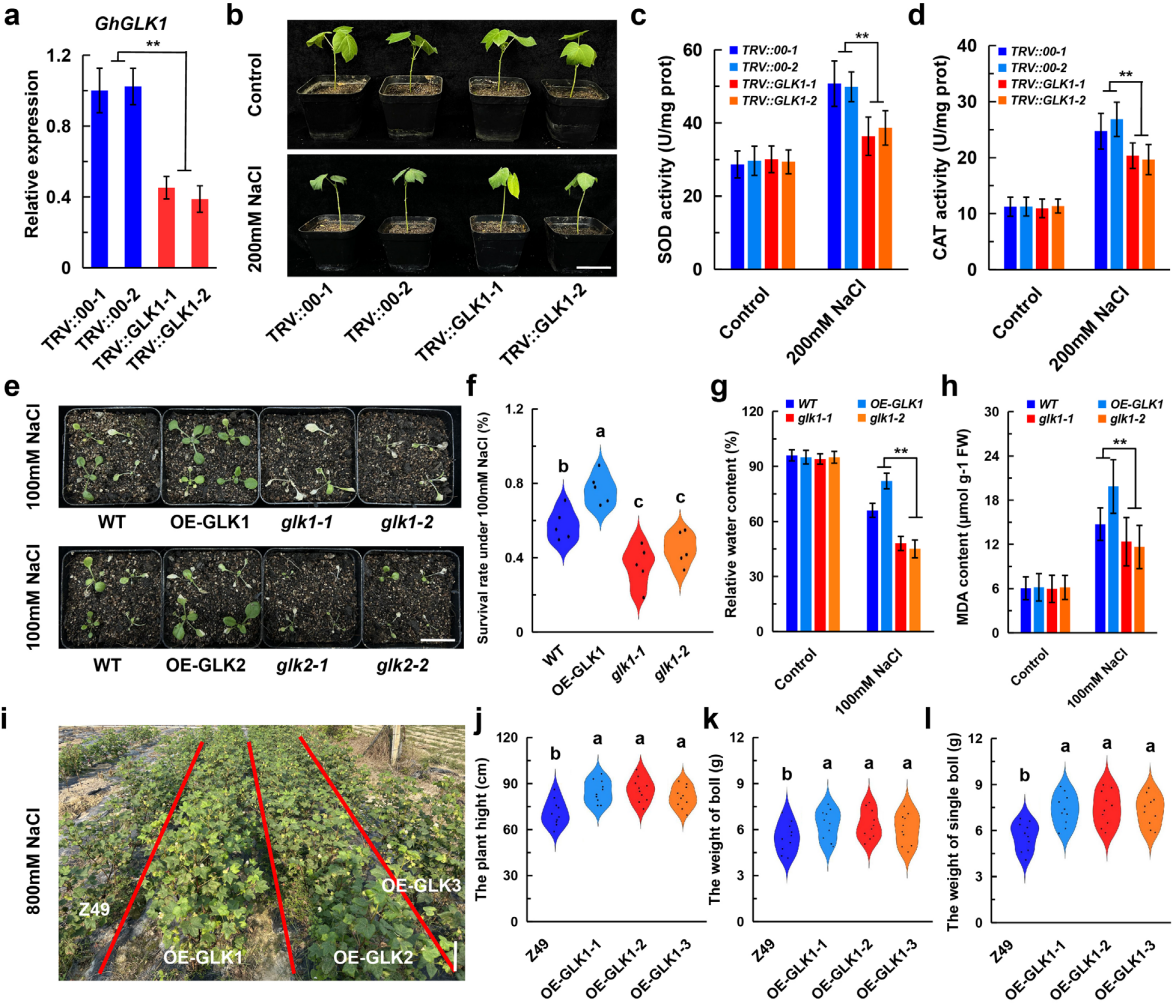
GhGLK1 enhances salt tolerance. (a) RT‐qPCR analysis of *GhGLK1* expression in TRV::00 and TRV::GhGLK1 cotton. Data represent mean ± SD (*n* = 3 replicates). **p < 0.01. Student's *t*‐test. (b) Phenotypic comparison of TRV::00 and TRV::GhGLK1 cotton under CK and 200 mM NaCl. Bar = 5 cm. (c, d) SOD and CAT activities in TRV::00 and TRV::GhGLK1 under CK and 200 mM NaCl. Data are presented as mean ± SD (*n* = 3 replicates). **p < 0.01. Student's *t‐*test. (e) Phenotypic comparison of WT, OE‐GLK1/2 and *glk1*/*2 Arabidopsis* under 100 mM NaCl. Bar = 3 cm. (f) Survival rates of WT, OE‐GLK1/2 and *glk1/2* under 100 mM NaCl. Data represent mean ± SD (*n* = 3 replicates). *p* < 0.05. ANOVA. (g) Relative water content of WT, OE‐GLK1/2 and *glk1/2* under 100 mM NaCl. Data represent mean ± SD (*n* = 3 replicates). *p* < 0.05. ANOVA. h. MDA content of WT, OE‐GLK1/2 and *glk1/2* under 100 mM NaCl. Data represent mean ± SD (*n* = 3 replicates). **p < 0.01. Student's *t‐*test. (i) Phenotypic comparison of Z49, OE‐GLK1‐1, OE‐GLK1‐2 and OE‐GLK1‐3 under NaCl treatment in field conditions. Bar = 15 cm. (j) Plant height, (k) boll weight (l) and single‐boll weight of Z49, OE‐GLK1‐1, OE‐GLK1‐2 and OE‐GLK1‐3 following NaCl treatment. Data represent mean ± SD (*n* = 3 replicates). *p* < 0.05. ANOVA.

VIGS‐mediated silencing significantly reduced GhGLK1 expression in TRV::GLK1 plants compared to TRV::00 controls (Figure 5a). After 7 days of silencing, plants were subjected to 200 mM NaCl treatment. By Day 12, TRV::GLK1 plants exhibited severe wilting earlier than TRV::00 (Figure 5b). Additionally, SOD (Figure 5c) and CAT (Figure 5d) activities were significantly lower in TRV::GLK1 plants, indicating heightened oxidative stress susceptibility. Interestingly, while GhBGH2 negatively regulated salt tolerance, GhGLK1 functioned as a positive regulator.

To further assess GhGLK1 function, *Arabidopsis* lines overexpressing *GLK1*/2 and *glk1/glk2* mutants were subjected to 100 mM NaCl at the seedling stage. Overexpression lines exhibited significantly greater salt tolerance compared to controls and glk1/2 mutants (Figure 5e). Relative survival rate (Figure 5f) and relative water content (Figure 5g) were also significantly higher in overexpressing lines, whereas MDA levels were lower, indicating reduced oxidative damage (Figure 5h).

Through *Agrobacterium*‐mediated transformation, three overexpression lines (OE‐GLK1‐1, OE‐GLK1‐2, OE‐GLK1‐3) were generated (Figure S4). Under field conditions, 800 mM NaCl treatment for 3 weeks resulted in severe dwarfing and leaf chlorosis in control plants (Z49) (Figure 5i,j). However, boll weight per plant and total boll weight were significantly higher in overexpression lines than in Z49, demonstrating enhanced salt tolerance in transgenic cotton (Figure 5k,l).

### 3.6 GhBGH2 Inhibits the Transcription of Salt‐Related Genes by Interacting With the GhGLK1 Transcriptional Activation Domain

Transcriptome sequencing of TRV::00 and TRV::GhGLK1 plants after salt stress, followed by GO/KEGG pathway enrichment, identified numerous stress‐associated pathways. These included ‘response to oxidative stress’, ‘response to stress’ and ‘regulation of response to salt stress’ in KEGG, alongside the ‘MAPK signaling pathway’, ‘ABC transporters’ and various amino acid metabolism pathways in GO (Figure S5a,b). Co‐expression analysis using the Cotton Multiomics Database (CottonMD, https://yanglab.hzau.edu.cn/CottonMD/) further supported GhGLK1 involvement in salt stress responses (Figure S5c).

**FIGURE 6:**
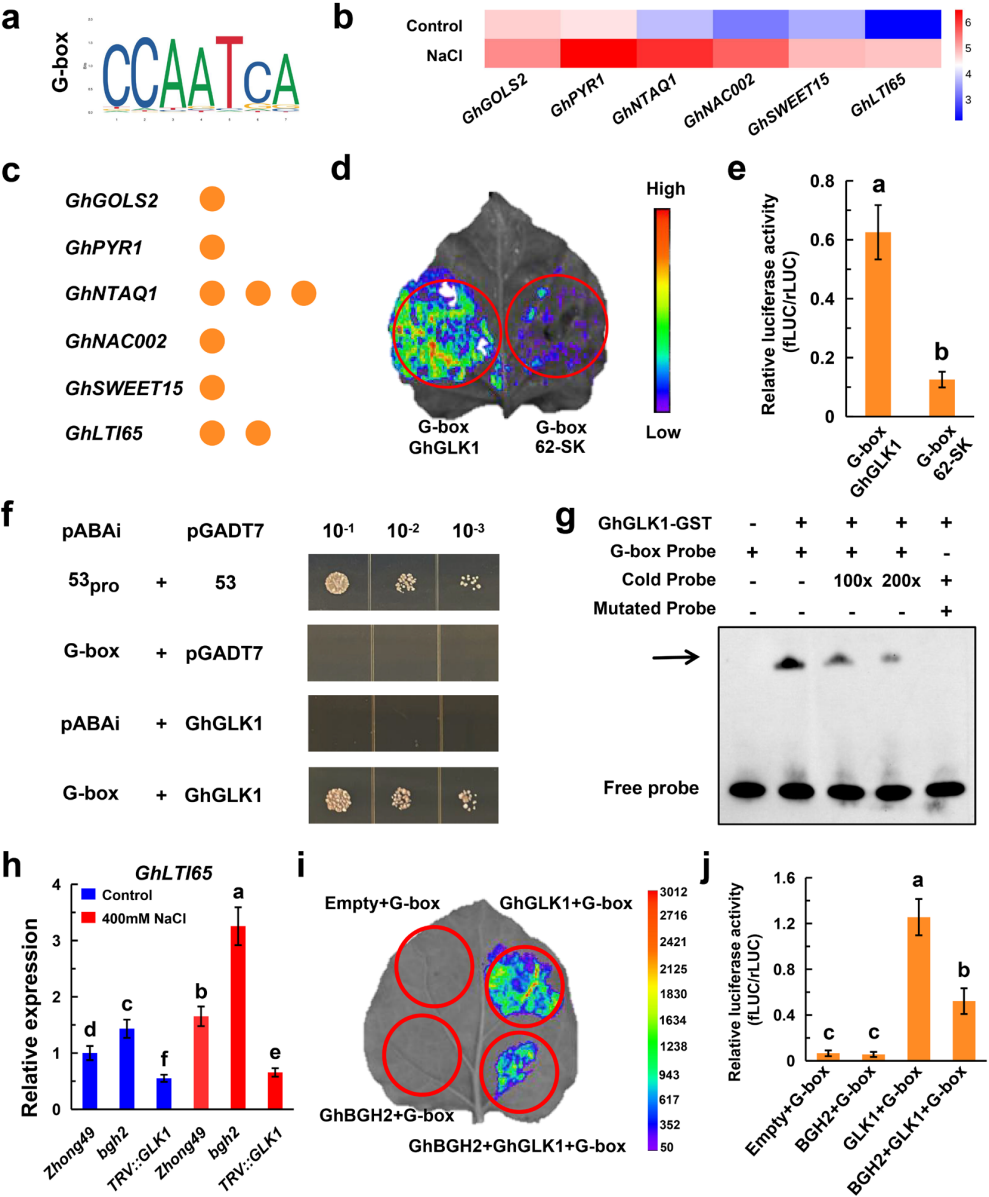
GhBGH2 interacts with the transcriptional activation domain of GhGLK1 to repress the transcription of downstream genes. (a) Yeast two‐hybrid assay confirming the interaction between GhBGH2 and GhGLK1. Serial dilutions (1×, 10× and 100×) of transformed yeast cells were spotted onto DDO and QDO solid media. ‘Empty’ denotes the AD/BD empty vector control. (b) Heatmap showing the expression levels of six candidate genes (*GhGOLS2*, *GhPYR1*, *GhNAC002*, *GhSWEET15*, *GhLTI65* and *GhNTAQ1*) under control and 200 mM NaCl conditions. (c) The number of G‐box elements in the promoter regions of the candidate genes, with each orange circle representing one G‐box. (d, e) Transient expression assay demonstrating that GhGLK1 binds to the G‐box element. Representative images of *N. benthamiana* leaves are shown in (d), while (e) presents the quantitative analysis of luminescence intensity. Data represent the mean ± SD (*n* = 3 replicates). **p < 0.01. Student's *t*‐test. (f) Yeast one‐hybrid assay verifying GhGLK1 binding to the G‐box element. The ABAi system was used, where the G‐box sequence was cloned into pABAi, and GhGLK1 was cloned into pGADT7. *53pro* and *53* served as positive controls. Cells were diluted (1×, 10× and 100×) in SD/−Leu media. (g) EMSA results showing GhGLK1‐GST binding to a G‐box‐containing DNA probe and a mutated probe. A cold probe was used as a negative control. (h) RT‐qPCR analysis of *GhLTI65* transcript levels in Z49, *bgh2* and TRV::GLK1 plants. Data represent the mean ± SD (*n* = 3 replicates). *p* < 0.05. ANOVA. (i, j) Transient expression assay of Empty+G‐box, GhBGH2 + G‐box, GhGLK1 + G‐box, and GhBGH2 + GhGLK1 + G‐box constructs. Representative *N. benthamiana* leaf images are shown in (i), and quantitative luminescence intensity data are in (j). Data represent the mean ± SD (*n* = 3 replicates). *p* < 0.05. ANOVA.

Previous studies indicate that GLK transcription factors bind G‐box elements in gene promoters (Figure 6a). Screening of salt‐tolerance genes—*GhGOLS2* (Takahashi et al. 1994; Taji et al. 2002; Wang, Nie, et al. 2023), *GhPYR1* (Santiago et al. 2009; Wang, Zhang, et al. 2023), *GhNAC002* (He et al. 2005; Wang et al. 2024), *GhSWEET15* (Fan et al. 2023; Huang et al. 2024), *GhLTI65* (Msanne et al. 2011; Wang, Nie, et al. 2023) and *GhNTAQ1* (Vicente et al. 2019)—revealed the presence of G‐box motifs within 2 kb upstream of their ATG start sites (Figure 6b). Notably, *GhGOLS2*, *GhPYR1*, *GhNAC002* and *GhSWEET15* contain one G‐box, whereas *GhLTI65* and *GhNTAQ1* harbour two and three, respectively (Figure 6c). In addition, the homologous genes of these genes were induced by salt stress in the Arabidopsis database and participated in the stress response (Figure S6).

A dual‐luciferase assay confirmed that GhGLK1 activates transcription through G‐box elements. Infection of 62‐SK‐GhGLK1 with 3 × G‐box‐LUC significantly increased LUC fluorescence and the LUC/REN ratio compared to controls (Figure 6d,e). Yeast one‐hybrid assays further demonstrated that strains carrying pGADT7‐GhGLK1 and 3 × G‐box‐pAbAi plasmids grew on SD/−Leu medium supplemented with 140 ng·mL^−1^ ABA, whereas strains with the empty pGADT7 vector failed to grow, confirming direct GhGLK1–G‐box binding (Figure 6f).

Electrophoretic mobility shift assays (EMSA) provided additional validation, showing that GhGLK1‐GST specifically binds to the G‐box motif (CCAATC). Competitive cold probes disrupted this interaction, while a mutated probe failed to bind GhGLK1‐GST (Figure 6g). RT‐qPCR analysis of *GhLTI65* expression in Z49, *bgh2* and TRV::GLK1 plants revealed that *GhLTI65* was upregulated in *bgh2* and downregulated in TRV::GLK1, particularly under salt stress (Figure 6h). Given that GhGLK1 expression is elevated in *bgh2* mutants, the observed upregulation of *GhLTI65* in *bgh2* likely results from GhBGH2‐GhGLK1 interactions.

Finally, to determine whether GhBGH2 affects the transcriptional activation of GhGLK1 on downstream genes, a dual‐luciferase assay was conducted using GhBGH2, GhGLK1 and the G‐box element. Luciferase activity indicated that GhBGH2 inhibits the transcriptional activation of *GhGLK1*, reducing expression of salt‐responsive genes (Figure 6i,j).

### 3.7 Haplotype Analysis in *GhGLK1* and Its Role in Salt Tolerance

**FIGURE 7:**
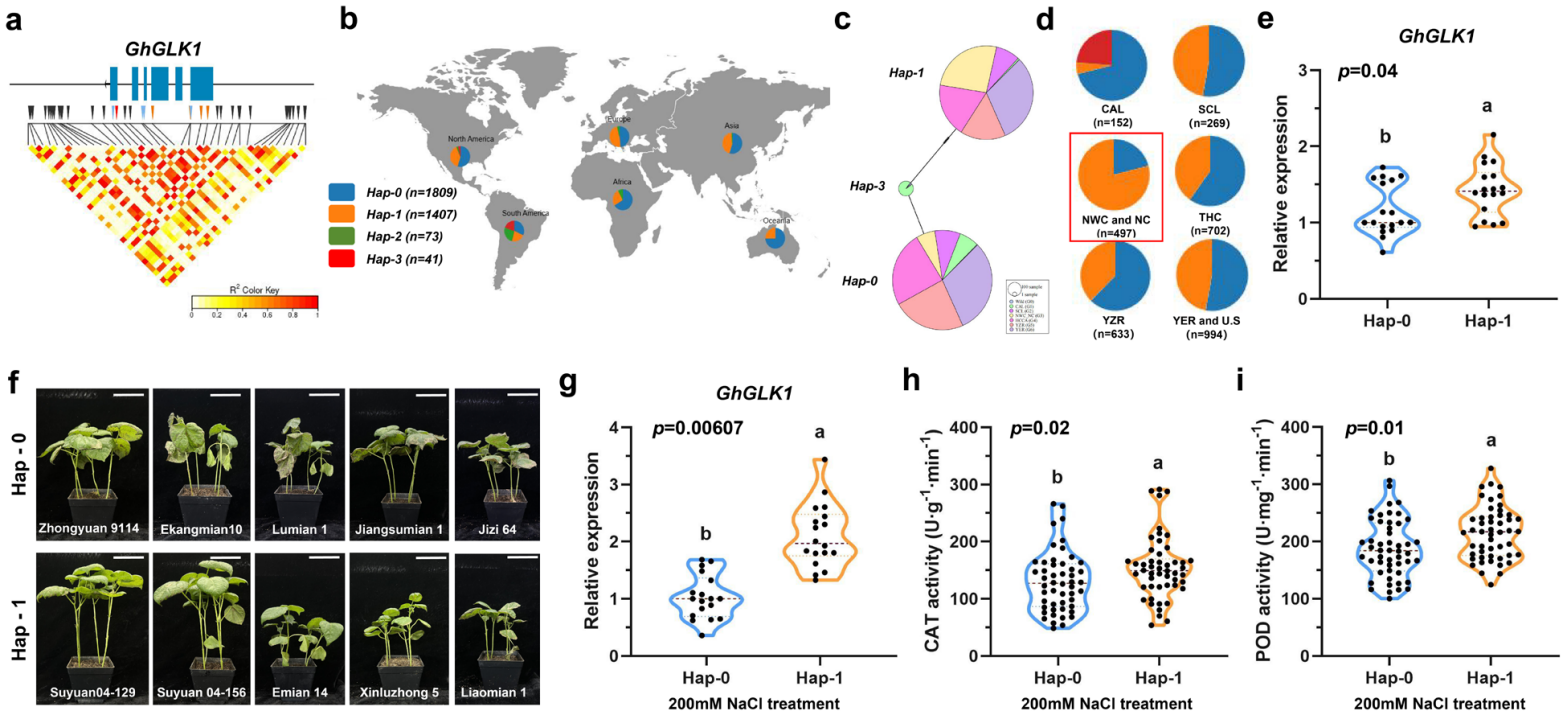
Haplotype analysis of *GhGLK1* suggests its role in salt tolerance. (a) SNP variation analysis in cotton accessions, identifying 39 SNPs within the 2‐kb upstream and downstream regions and coding sequence of *GhGLK1*, including 11 SNPs in the coding region. Among these, four were missense mutations, and four were located in splice sites or intronic regions. (b) Haplotype analysis of SNP variations across different regions. (c) Geographic distribution of haplotypes in cotton accessions, with colours representing different regions: Central American landraces (CAL), South China landraces (SCL), Northwest China (NWC), North China (NC), three historical Chinese cotton regions (THC), Yangtze River region (YZR), Yellow River region (YER) and the United States. (d) Proportional distribution of haplotypes across regions. (e) RT‐qPCR analysis of *GhGLK1* transcript levels in *Hap‐0* and *Hap‐1* before NaCl treatment. Data represent the mean ± SD (*n* = 18). *p* < 0.05. ANOVA. (f) Phenotypic comparison of *Hap‐0* and *Hap‐1* under 200 mM NaCl treatment. Bar = 3 cm. (g) RT‐qPCR analysis of *GhGLK1* transcript levels in *Hap‐0* and *Hap‐1* after NaCl treatment. Data represent the mean ± SD (*n* = 18). *p* < 0.05. ANOVA. (h, i) CAT and POD enzyme activities in *Hap‐0* and *Hap‐1* under 200 mM NaCl treatment. Data represent the mean ± SD (*n* = 50 replicates). *p* < 0.05. ANOVA.

To investigate genetic variation in *GhGLK1* and identify alleles associated with salt tolerance, we analysed its haplotypes using the CottonMD database, which includes 4180 cotton accessions from diverse global regions. Within the 2‐kb upstream and downstream regions and the coding sequence of *GhGLK1*, we identified 39 SNPs, of which 11 were located in coding regions, including four missense mutations and four within splice sites or intronic regions (Figure 7a). Haplotype analysis grouped these SNP variations into four major types (Figure 7b). *Hap‐0* (*n* = 1697) and *Hap‐1* (*n* = 1516) were the predominant haplotypes in *Gossypium hirsutum* , while *Hap‐2*(0) and *Hap‐3 (*Tachibana et al. 2024) were rare (*n* = 3). In contrast, *Hap‐0*, *Hap‐2* and *Hap‐3* collectively accounted for 99.4% of *G. barbadense* , with *Hap‐1* comprising only 0.6% (Figure S7a).

Further analysis revealed that *Hap‐3* in *G. hirsutum* was primarily found in wild species and landraces from China and the United States, whereas cultivated accessions were dominated by *Hap‐0* and *Hap‐1* (Figure 7c). Notably, in the G3 group comprising accessions from arid and semi‐arid regions of northern and northwestern China, the distribution of these haplotypes was significantly skewed, with *Hap‐1* accounting for 78.8% and *Hap‐0* for 21.2% (Figure 7d). This suggests that superior *GhGLK1* haplotypes may have been selectively utilised in breeding programmes for drought‐prone environments. Based on these findings, we selected accessions from the G3 group carrying different haplotypes for further analysis. Sequencing of five randomly chosen materials from *Hap‐0* and *Hap‐1* revealed a CAG‐C deletion mutation in the Hap1 accessions (Figure S7b). RT‐qPCR further confirmed that *Hap‐1* was associated with a marked increase in *GhGLK1* expression (Figure 7e). Salt tolerance assays under 200 mM NaCl treatment indicated that *Hap‐1* accessions exhibited significantly greater salt tolerance compared to *Hap‐0* (Figure 7f). Upon NaCl treatment, Hap1 accessions showed a significantly greater upregulation of *GhGLK1* expression (Figure 7g). Additionally, enzymatic assays on 10 randomly selected plants demonstrated that Hap1 accessions had significantly higher SOD (Figure 7h) and CAT (Figure 7i) activity compared to the reference group, reinforcing their enhanced salt tolerance.

**FIGURE 8:**
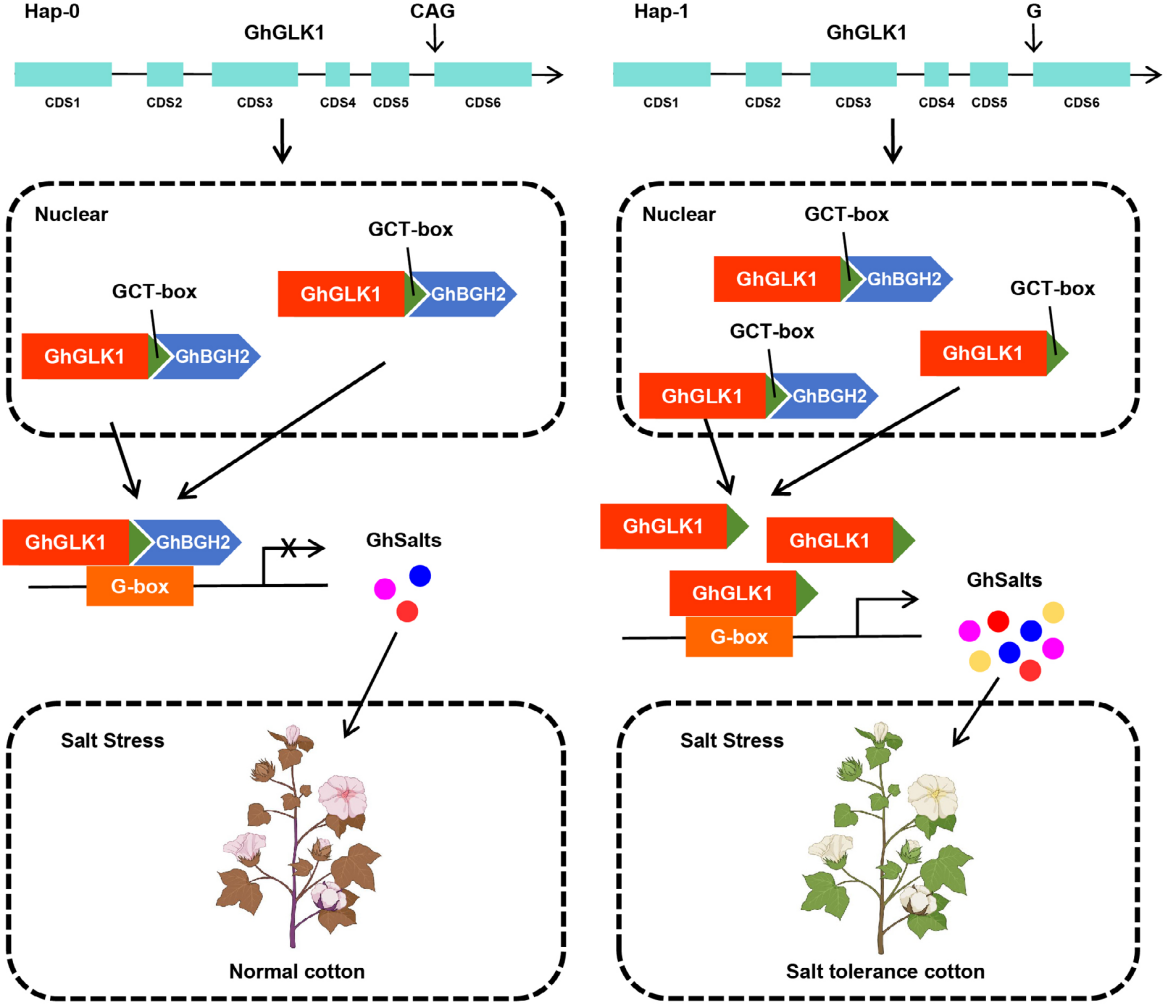
The GhBGH2‐GhGLK1 regulation module mediates salt tolerance in cotton. Under normal conditions, GhBGH2 interacts with the C‐terminal GCT‐box of GhGLK1, inhibiting its transcriptional activation of salt tolerance‐related target genes, resulting in their low expression. When *GhBGH2* is knocked out, this repression is lifted, allowing GhGLK1 to upregulate downstream salt tolerance genes, enhancing salt stress resistance.

In summary, we propose a regulatory model in which *GhBGH2* interacts with the C‐terminal GCT‐box of *GhGLK1* under normal conditions, repressing its transcriptional activation of downstream salt‐tolerance genes, which remain at low expression levels. Upon *GhBGH2* knockout, this repression is lifted, allowing GhGLK1 to activate a suite of salt‐tolerance‐related genes, thereby enhancing stress resistance. Additionally, our genetic analysis identified a deletion mutation upstream of CDS6 that may alter the cleavage recognition site sequence, ultimately leading to increased *GhGLK1* expression and subsequent activation of salt‐tolerance genes (Figure 8).

## 4 Discussion

GLKs were initially named after the Golden2 (G2) protein in maize, which was first identified as a regulator of chloroplast development. These transcription factors share homology with ARR‐B (arabidopsis response regulator‐B) in Arabidopsis and PSR1 (phosphate starvation response 1) in *Chlamydomonas* (Riechmann et al. 2000). Previous studies have shown that GLKs play a crucial role in chloroplast development by upregulating the expression of *σ* factors (SIG), activating plastid‐encoded RNA polymerase (PEP). This activation enhances the transcription of plastid‐encoded genes associated with photosynthesis, including those for Photosystem I (PSI) and Photosystem II (PSII), promoting chloroplast formation and function. Salt stress primarily affects plant root systems through osmotic stress, triggering a cascade of physiological and biochemical responses that impair overall growth and development. At the photosynthetic level, salt stress disrupts electron transport, affects stomatal conductance and reduces the photosynthetic rate, ultimately inhibiting plant growth (Diego et al. 2003). However, the full potential of photosynthetic adaptation under salt stress remains underexplored. Prior studies have demonstrated that photosynthetic efficiency can be improved under saline conditions by optimising electron transport between photosystems, utilising beneficial microbial inoculations and modifying root architecture (Zahra et al. 2022). A promising strategy to enhance salt tolerance is the overexpression of photosynthesis‐related genes, which could help stabilise photosynthetic capacity, mitigate the decline in photosynthetic rate and regulate stomatal function under stress conditions (Wang et al. 2024).

In this context, GLK1 may play a pivotal role in regulating light‐responsive genes, thereby enhancing photosynthesis and promoting salt tolerance through the upregulation of salt‐responsive genes. Additionally, we propose that GLK1 could be involved in balancing photosynthesis and stress adaptation. Under stress conditions, despite the suppression of plant growth, photosynthetic activity (the ‘source’) continues. This suggests a breakdown in the balance between the source and the ‘sink’, where the latter is unable to effectively utilise the assimilates supplied by the source. Understanding the role of GLK1 in this source–sink interaction may provide new insights into stress‐adaptive regulatory mechanisms. Besides, BR enhances the adaptability of plants to adversities such as drought, salinity, low temperature, high temperature, pests and diseases by regulating cell membrane stability, antioxidant system, osmoregulation, gene expression and other pathways. Previous references reported that GLK1 can be involved in light and BR signalling. Interestingly, BR also negatively regulates BGP activity through feedback inhibition (Tachibana et al. 2022). In this study, the knockdown of *bgh2* upregulated GLK1, which activated the BR pathway and participated in plant stress response.

Plants have evolved sophisticated mechanisms to perceive environmental stress, transmit signals intracellularly and adjust their growth accordingly. One major consequence of stress is the overproduction of reactive oxygen species (ROS), which is a double‐edged sword in plant biology (Gong et al. 2020). At low levels, ROS serve as signalling molecules that regulate growth, development and adaptation. However, under severe stress, ROS production increases dramatically, overwhelming antioxidant defence systems and disrupting cellular redox balance, ultimately leading to oxidative damage (Sachdev et al. 2021). Among ROS, hydrogen peroxide (H_2_O_2_) is a key signalling molecule involved in growth regulation and stress responses (He et al. 2021). Overexpression of *GhGLK1* in *Arabidopsis thaliana* has been shown to enhance tolerance to drought and cold stress, with elevated H_2_O_2_ levels detected in transgenic plants following stress treatment (Liu et al. 2021). In the present study, trypan blue staining indicated that the *bgh2* mutant exhibited greater ROS scavenging capacity and sustained less damage under salt stress. This suggests that *GhGLK1* may modulate ROS homeostasis, enhancing resistance to multiple abiotic stresses.

Plants frequently undergo alternative splicing to enhance adaptability to environmental stress (Lam et al. 2022; Punzo et al. 2020; Yang et al. 2022). Our haplotype analysis revealed that Hap1 carries a deletion mutation (CAG‐C) upstream of CDS6. Although this mutation does not disrupt the canonical splice recognition site (GU‐AG), it induces a frameshift in the adjacent sequence, potentially leading to alternative splicing (Marasco and Kornblihtt 2023). This modification may influence CDS6 transcript levels and, consequently, GhGLK1‐mediated transcriptional regulation of downstream stress‐response genes.

## Author Contributions

P.W., H.G. and H.C.: conceptualisation. P.W., H.G.: design and revision. P.W., J.W. and W.L.: validation. J.W., W.L., C.L., X.S., X.C. and L.Z.: investigation. X.X., W.G., H.S.: formal analysis. X.S., H.S.: software. P.W.: writing original draft preparation. P.W., H.C. and H.G.: review and editing. P.W., H.C., B.M. and H.G.: funding acquisition. All authors have read and agreed to the published version of the manuscript. All authors read and approved the final manuscript.

## Conflicts of Interest

The authors declare no conflicts of interest.

## Supporting information


**Data S1:** pbi70300‐sup‐0001‐supinfo.docx.

## Data Availability

The data that support the findings of this study are available on request from the corresponding author. The data are not publicly available due to privacy or ethical restrictions.
