# Supplementary material for: A GhBGH2‐GhGLK1 Regulatory Module Mediates Salt Tolerance in Cotton
Source: Plant Biotechnol J. 2025 Aug 6;23(11):5197–210. doi: 10.1111/pbi.70300 (PMC12576457; doi:10.1111/pbi.70300)
Supplement: Supplementary file 1 — Data S1: pbi70300‐sup‐0001‐supinfo.docx. [file PBI-23-5197-s001.docx]

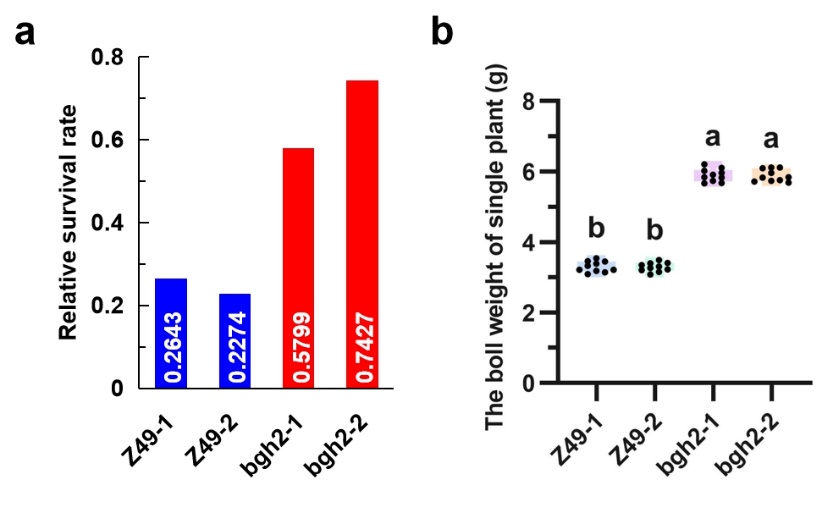


**Figure S1 Field survival rates of Z49 and *bgh2* after 0.4% NaCl treatment. a.** Resistance levels were classified as follows: LP ≥ 90 (high resistance), 75–89.9 (salt resistance), 50–74.9 (salt tolerance), and <50 (salt sensitivity). b. The boll weight of single plant. Data represent the mean ± S.D (n = 10 replicates). P < 0.05. ANOVA.


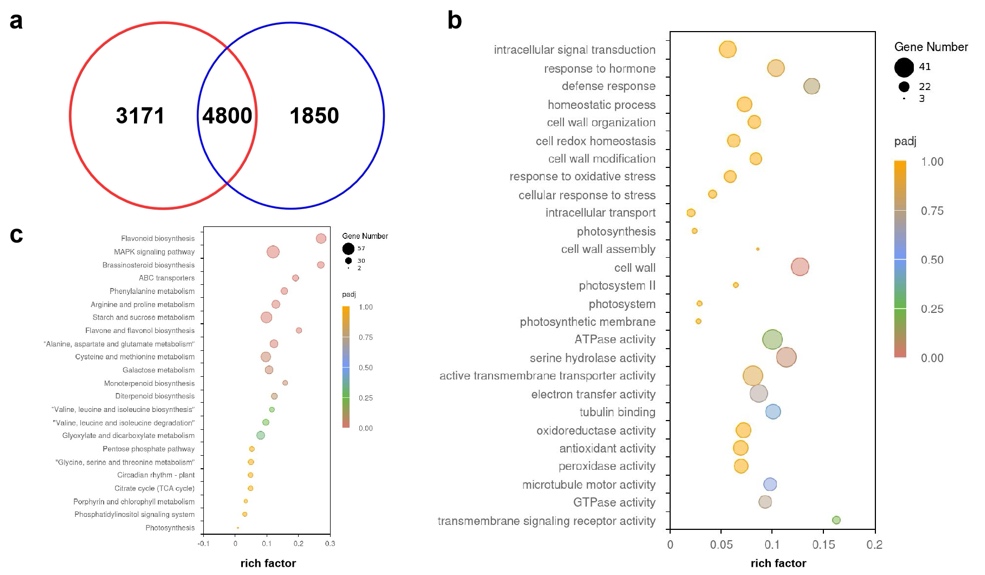


**Figure S2 GO and KEGG enrichment analysis for salt tolerance pathways and candidate gene identification. a**. Venn diagrams displaying the overlap of DEGs identified in *Z49 vs. bgh2-1* and *Z49 vs. bgh2-2s*. DEGs were defined by |Log2FoldChange| ≥ 1 and *P* ≤ 0.05. **b.** Salt-related pathways enriched in KEGG analysis. **c.** Salt-related pathways enriched in Gene Ontology (GO) analysis.


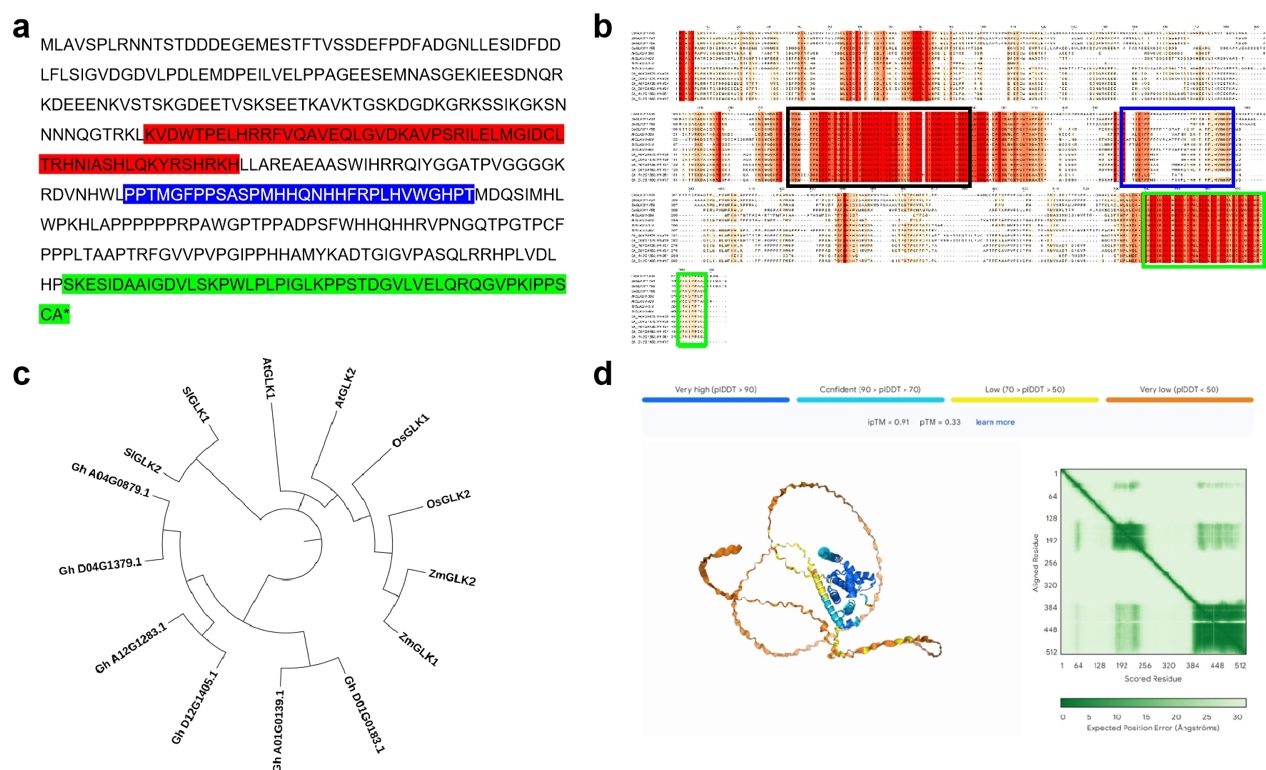


**Figure S3 GhGLK1 structure, evolution, and protein prediction. a**. Protein domain architecture of GhGLK1, consisting of an N-terminal acidic domain, a DNA-binding domain, a proline-rich region, and a C-terminal GCT-box-containing transcriptional activation domain **b.** Comparative sequence analysis of GhGLK1/2 homologs in *Arabidopsis*, maize, rice, and tomato reveals conserved DNA-binding and C-terminal domains. **c.** Phylogenetic tree of GLK1 in *Arabidopsis thaliana* and other species (*Gh, Gossypium hirsutum; At, Arabidopsis thaliana; Os, Oryza sativa; Sl, Solanum lycopersicum; Zm, Zea mays*). **d.** AlphaFold3-predicted protein interaction model of GhBGH2 and GhGLK1, highlighting their interaction region in blue. The local distance difference test (pLDDT) was used as a confidence measure.


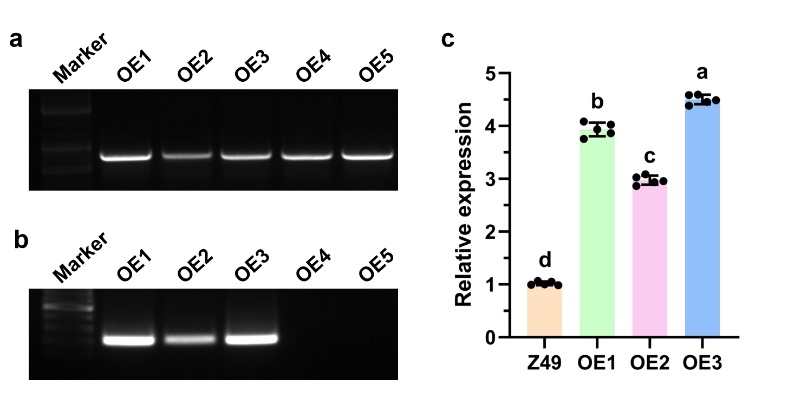


**Figure S4 Molecular identification of overexpression of OE-GLK1. a**. PCR identification. **b.** RT-PCR identification. **c.** RT-qPCR identification.


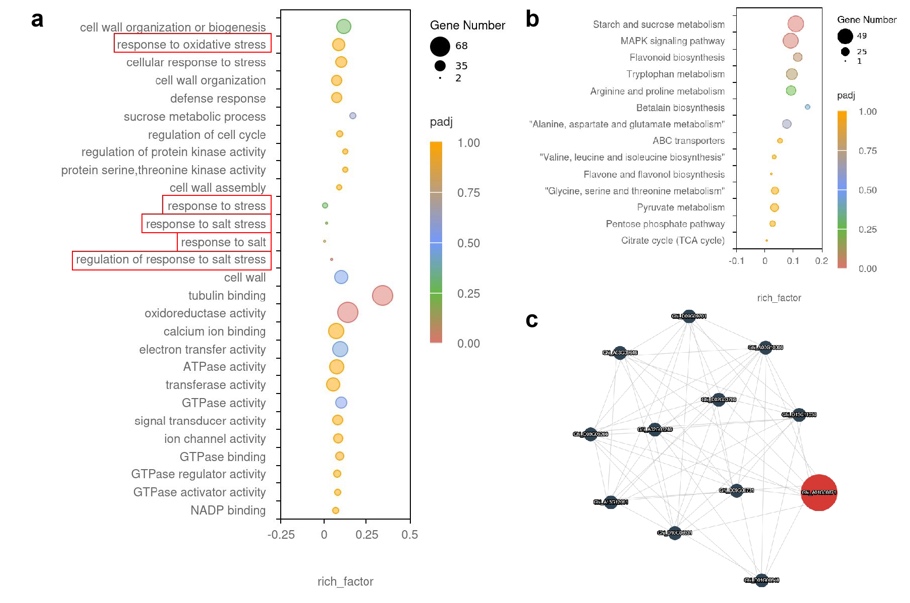


**Figure S5 GO and KEGG enrichment analysis for salt tolerance-related pathways and candidate gene identification. a-b**. Venn diagrams displaying the overlap of DEGs identified in *TRV::00 vs. TRV::GLK1*. DEGs were defined by |Log2FoldChange| ≥ 1 and *P* ≤ 0.05. **c.** Co-expression analysis of GhGLK1 using CottonMD (https://yanglab.hzau.edu.cn/CottonMD.1).


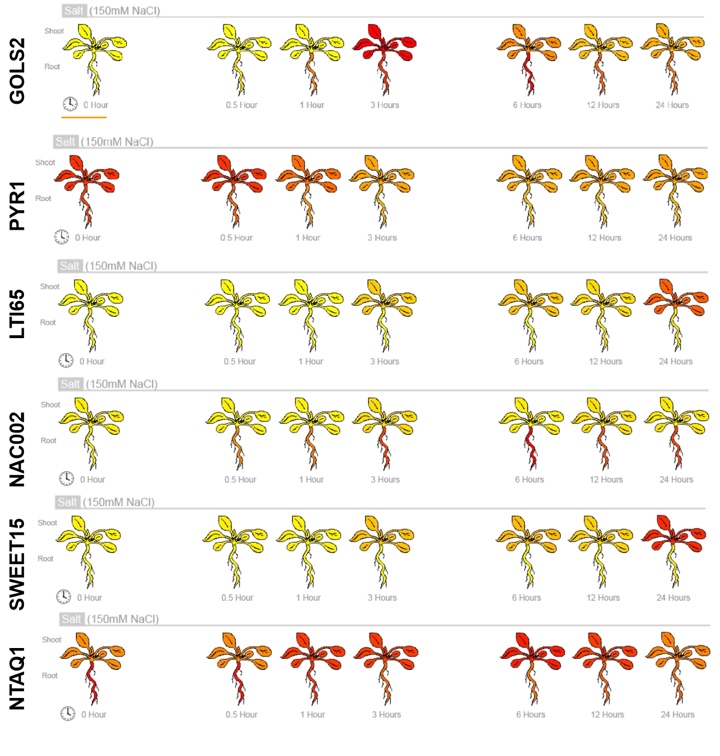


**Figure S6 Heatmap of GhGLK1-regulated candidate genes under NaCl stress, analyzed using the TAIR database** (https://www.arabidopsis.org/).


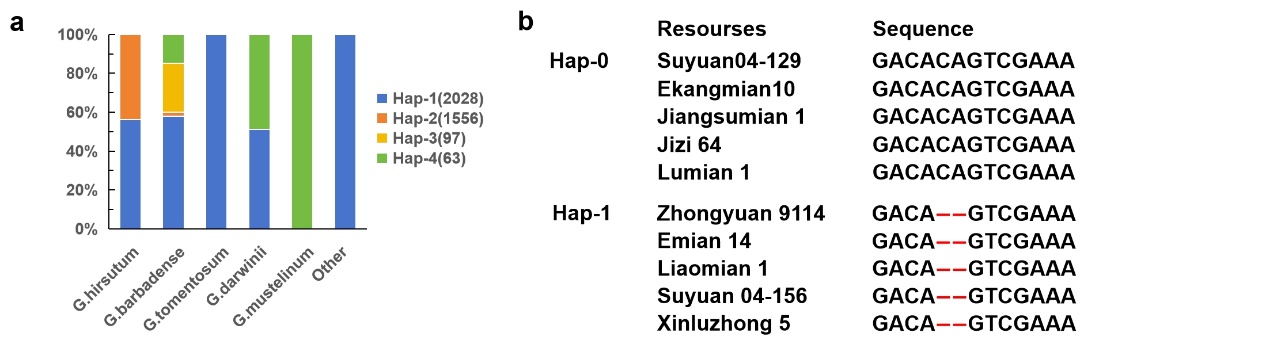


**Figure S7 Haplotype analysis of *GhGLK1*.** a. Proportional distribution of GhGLK1 haplotypes across different cotton species. **b.** Sequencing analysis of *Hap-0* and *Hap-1*, revealing base deletions upstream of CDS6.
